# Three-Year Study of Markers of Oxidative Stress in Exhaled Breath Condensate in Workers Producing Nanocomposites, Extended by Plasma and Urine Analysis in Last Two Years

**DOI:** 10.3390/nano10122440

**Published:** 2020-12-06

**Authors:** Daniela Pelclova, Vladimir Zdimal, Martin Komarc, Jaroslav Schwarz, Jakub Ondracek, Lucie Ondrackova, Martin Kostejn, Stepanka Vlckova, Zdenka Fenclova, Stepanka Dvorackova, Lucie Lischkova, Pavlina Klusackova, Viktoriia Kolesnikova, Andrea Rossnerova, Tomas Navratil

**Affiliations:** 1 Department of Occupational Medicine, First Faculty of Medicine, Charles University in Prague and General University Hospital in Prague, Na Bojisti, 128 00 Prague, Czech Republic; stepanka.vlckova@vfn.cz (S.V.); zdenka.fenclova@lf1.cuni.cz (Z.F.); lucie.lischkova@vfn.cz (L.L.); pavlina.klusackova@vfn.cz (P.K.); viktoriia.kolesnikova@vfn.cz (V.K.); 2 Institute of Chemical Process Fundamentals CAS, Rozvojova 1/135, 165 02 Prague, Czech Republic; zdimal@icpf.cas.cz (V.Z.); schwarz@icpf.cas.cz (J.S.); ondracek@icpf.cas.cz (J.O.); ondrackova@icpf.cas.cz (L.O.); kostejn@icpf.cas.cz (M.K.); 3 Institute of Biophysics and Informatics, First Faculty of Medicine, Charles University and General University Hospital in Prague, Salmovska, 120 00 Prague, Czech Republic; martin.komarc@lf1.cuni.cz or; 4 Faculty of Physical Education and Sport, Charles University and General University Hospital in Prague, José Martího 31, 162 52 Prague, Czech Republic; 5 Department of Machining and Assembly, Department of Engineering Technology, Department of Material Science, Faculty of Mechanical Engineering, Technical University in Liberec, Studentska 1402/2, 461 17 Liberec, Czech Republic; stepanka.dvorackova@tul.cz; 6 Department of Genetic Toxicology and Epigenetics, Institute of Experimental Medicine CAS, Videnska 1083, 142 20 Prague, Czech Republic; andrea.rossnerova@iem.cas.cz; 7 J. Heyrovský Institute of Physical Chemistry CAS, Dolejškova, 182 23 Prague, Czech Republic; Tomas.Navratil@jh-inst.cas.cz

**Keywords:** nanoparticles, oxidative stress, biomarkers, workers, controls, exhaled breath condensate, plasma, urine

## Abstract

Human data concerning exposure to nanoparticles are very limited, and biomarkers for monitoring exposure are urgently needed. In a follow-up of a 2016 study in a nanocomposites plant, in which only exhaled breath condensate (EBC) was examined, eight markers of oxidative stress were analyzed in three bodily fluids, i.e., EBC, plasma and urine, in both pre-shift and post-shift samples in 2017 and 2018. Aerosol exposures were monitored. Mass concentration in 2017 was 0.351 mg/m^3^ during machining, and 0.179 and 0.217 mg/m^3^ during machining and welding, respectively, in 2018. In number concentrations, nanoparticles formed 96%, 90% and 59%, respectively. In both years, pre-shift elevations of 50.0% in EBC, 37.5% in plasma and 6.25% in urine biomarkers were observed. Post-shift elevation reached 62.5% in EBC, 68.8% in plasma and 18.8% in urine samples. The same trend was observed in all biological fluids. Individual factors were responsible for the elevation of control subjects’ afternoon vs. morning markers in 2018; all were significantly lower compared to those of workers. Malondialdehyde levels were always acutely shifted, and 8-hydroxy-2-deoxyguanosine levels best showed chronic exposure effect. EBC and plasma analysis appear to be the ideal fluids for bio-monitoring of oxidative stress arising from engineered nanomaterials. Potential late effects need to be targeted and prevented, as there is a similarity of EBC findings in patients with silicosis and asbestosis.

## 1. Introduction

Despite the extensive production and use of nanoproducts, knowledge concerning the safety of their handling and use is lagging, and no biomarkers are currently used to monitor exposure in practice.

Several studies have searched for the most useful biomarkers of oxidative damage in humans [1,2,3,4,5], and it has been shown that no single marker may be satisfactory [6,7]. The need for nanotoxicology studies in humans is considered vital due to the potential damage to cells, organs, and organisms, and to a lack of human data, especially in occupational settings [8,9], where the exposure is the highest. Consumer product usage, such as sunscreen, may also be cause for concern, as absorption of nanoparticles through the skin was found [10,11]. The experimental data show that cell membranes with lipid bilayers are the primary target for nanoparticle attack, which may ultimately lead to cell death [12]. It has been shown that nanoparticle-induced oxidative stress and reactive oxygen species (ROS) produced can lead to inflammation, apoptosis, and genotoxicity. Additionally, a high accumulation of nanoparticles on the cell surface is a further trigger of cytotoxicity. Moreover, chronic, even low-dose exposures may induce more subtle changes and lead to disease initiation or progression [12]. Particularly, little is known about more complex mixtures of nanoparticles and their interactions with other factors, such as diet or physical load.

In our previous study in 2016, we have described and characterized, in detail, the workplace aerosols formed in the course of the production and handling of nanocomposites during all three main operations in workshops (welding, smelting, and machining) [13,14]. Eleven in vivo markers of oxidative stress and 12 markers of inflammation were analyzed in the exhaled breath condensate (EBC) of workers in the nanocomposites research plant [13,14]. Significant increases in the majority of pre-shift and post-shift markers of oxidation of lipids, nucleic acids and proteins in the EBC of workers were observed, compared to control subjects’ samples. Moreover, spirometry showed impairment in post-shift lung function.

For this follow-up study in 2017 and 2018, eight markers of oxidative stress were selected, including biomarkers derived from free radical oxidation of polyunsaturated fatty acids malondialdehyde (MDA), aldehydes C6–C12 (i.e., hexanal (C6), heptanal (C7), octanal (C8), nonanal (C9), decanal (C10), undecanal (C11) and dodecanal (C12)), and 8-*iso*-prostaglandin F2α (8-isoprostane). Markers of oxidation of nucleic acid bases, including 8-hydroxy-2-deoxyguanosine (8-OHdG), 8-hydroxyguanosine (8-OHG), and 5-hydroxymethyl uracil (5-OHMeU), were examined. Markers of oxidative stress related to proteins, including *o*-tyrosine (*o*-Tyr) and 3-nitrotyrosine (3-NOTyr), were examined.

To improve and enlarge the pilot study from 2016, we extended the analysis of markers to plasma and urine in the following two years, and, to the best of our knowledge, no study has yet focused on the analysis of oxidative stress markers across several bodily fluids of workers handling nanomaterials.

The control subjects in 2016 and 2017 provided samples in the morning only, whereas, in 2018, the control subjects’ examination was enriched by a second sampling in the afternoon, at a comparable time of the post-shift samples collection in the workers.

The first aim of this study was to find out whether ongoing exposure to workplace aerosol, with a relatively stable proportion of ultrafine particles under similar conditions, would show an increasing trend, due to a potentially cumulative effect, or a negative trend, due to a potential adaptation to chronic exposure. To achieve this, we have also compared the results to those from the subgroup of workers who participated repeatedly and who provided their EBC samples in all three years (2016, 2017, and 2018). Here, we focused both on chronic effects, using pre-shift sample collection, and the acute effects related to the last shift exposure, as both effects were observed in 2016.

Our second aim was to verify the practical usefulness of the noninvasive method of EBC collection successfully used in 2016 and to compare the results of the same markers, collected and analyzed in parallel, in plasma and urine over the two following years, 2017 and 2018. This aim was selected due to concerns about the methodology related to dilution of the EBC fluid, for which no parameter exists to control concentration [15].

Our third aim was to select the most representative fluids and markers for bio-monitoring of the workers handling nanomaterials in order to monitor exposure and prevent unwanted late effects from appearing in experimental studies.

## 2. Materials and Methods

### 2.1. Operations Description

The research plant studied investigates and produces newly designed nanocomposite materials with similar characteristics to steel, specifically its low expansion with increasing temperature, its hardness and surface durability [16]. The workers would usually perform the following operations: welding of metal materials, smelting of mixtures containing nano-additives (both in workshop 1), and machining of the produced nanocomposite (workshop 2). These procedures and workplace aerosol measurements have already been documented in detail in our previous papers, evaluating studies carried out in 2016 [13,17,18].

The daily operations of the researchers had not substantially changed during the years; however, the monitoring days differed throughout each year: in 2016, exposure included all three operations (welding, smelting and machining). In 2017, only one (machining, evaluated as the highest-exposure operation in 2016), and two operations in 2018 (welding and machining).

Machining operations differed over time as the research aims evolved. For the monitoring day in 2016, there were three geopolymer samples, and two samples contained epoxide resin with nanoSiO_2_ filler. In 2017, out of six samples with epoxide resin, three contained nanoSiO_2_ filler, and three had filler from recycled carbon fibers diameter 7–8 µm, length up to 100 µm. In the year 2018, all eight samples consisted of epoxide resin with nanoSiO_2_ filler. As in previous years, the grinders used pink corundum discs containing Al_2_O_3_, Cr_2_O_3_, and Fe_2_O_3_ [13,17,18]. The technology of welding on metal surfaces used in the plant did not change during the three years.

### 2.2. Subjects

In a follow-up of research workers at the nanocomposite production plant, for whom only EBC was examined in 2016 [13], the markers of oxidative stress were analyzed in the EBC, plasma and spot urine samples collected in 2017 and 2018.

In 2017, there were 20 workers and 20 control subjects examined in parallel in the morning at approximately the same time as the researchers, and the workers were also examined post-shift.

In 2018, there were 21 workers and 18 control subjects examined twice, both in the morning and in the afternoon, at about the same time as the workers.

Among the workers examined, 17 subjects participated in both studies organized in 2017 and 2018.

The control subjects came from the same location and were not employed in dusty workplaces. Characteristics of all subject groups are presented in Table 1.

#### Questionnaire

All subjects answered questions focusing on their occupational, personal, and environmental histories.

Occupational history included the type of exposure in the workshops (welding, smelting, machining), materials used, length in years of exposure to nanoparticles, length of usual daily exposure, and latency since the end of the last shift. The questions also focused on the type of personal equipment used during the work in the workshops.

The personal history contained 20 questions: age, gender, alcohol intake (occasionally/never), smoking (yes/no, packyears), regular physical activity (more than 30 min daily), medication, diseases (including those described in the examination in 2016, such as hypothyroidism, hypertension, dyslipidemia, allergic rhinitis, bronchial asthma, other allergies, dysrhythmia, hyperbilirubinemia), symptoms (cough, dyspnea, the common cold), time and description of the last food ingested, and smoking within the last two hours (yes/no).

All subjects had a physical examination in an ambulatory room in another building. Exclusion criteria for all subjects were: recent fever, congenital heart disease, myocarditis, myocardial infarction, history of tuberculosis, interstitial lung disease, and cancer. The physical examination was followed by the collection of EBC, blood and urine, and spirometry.

Nanocomposites workers provided biological samples both pre-shift (i.e., before workshop exposure) and post-shift (i.e., within 1 h after spending 2.5–3.5 h in the workshop). As this represented only a part of their 8 h shift, they then continued to work in their office rooms.

The pre-shift samples were intended to reflect chronic effect(s) on the workers due to their long-term exposures to nanomaterials. The comparison of the pre-shift and post-shift samples was intended to reflect the acute effect(s) related to exposure during their shift.

All subjects gave their written consent for inclusion before participating in the study. This study was conducted in accordance with the Declaration of Helsinki, and the protocol was approved by the Ethics Committee of the General University Hospital in Prague and First Faculty of Medicine, Charles University (dates of approval: 19 March 2015; project identification code 9/15 Grant VES 2015 AZV 1. LFUK; and 16 March 2017; project identification code, 2/17 Grant GA CR-VFN).

### 2.3. Workplace Aerosol Measurements

The measurement of aerosol exposure at the workplace was described in detail in previous reports [13,17,18]. Therefore, only a short summary of our approach is given here.

The same sampling strategy has been applied in each of the three years (2016–2018). Both online and off-line instruments were placed at the same locations each year, and the same sampling parameters were retained, including flow rates and length of sampling tubes, among others. Before the start of nanoparticle production in each workshop, the background conditions were recorded for a substantial amount of time, usually overnight. Then, all instruments sampled continuously throughout the duration of nanoparticle production in the workshops.

To determine the average mass concentration of the aerosols during individual operations and to assess the chemical composition of different size fractions, a Berner low-pressure cascade impactor (BLPI, HAUKE GmbH, Gmunden, Austria) was used, which separates particles based on their inertia into 10 size fractions, covering the size range from 25 nm to 13.6 µm [19]. Gravimetric analysis of the impactor substrates was carried out using an M5P balance (Sartorius GmbH, Göttingen, Germany, 1 µm resolution).

For online determination of the particle size distributions, the two aerosol spectrometers were used to cover the size range of particles from about 10 nm to 10 µm in diameter. A scanning mobility particle sizer (SMPS 3936 L, TSI Inc., Shoreview, MN, USA), which sizes particles according to their electrical mobility, was utilized to cover the submicron size range (up to approximately 600 nm). The aerodynamic particle sizer (APS 3321, TSI Inc., Shoreview, MN, USA), which sizes particles based on their inertia, was applied to cover mainly the super-micron size range (from approximately 550 nm upwards). The time resolution of both instruments was set to 5 min. In order to follow the faster processes, a condensation particle counter (CPC, TSI Inc., Shoreview, MN, USA) was also used, recording total particle number concentration in the submicron range, with a time resolution of 1 s. In order to monitor the spatial distribution of the particles, three optical particle sizers (OPS 3330, TSI Inc., Shoreview, MN, USA) have also been used, sizing particles based on their optical properties, covering an approximate size range from 300 nm to 10 µm.

In one part of the substrates, the level of water-soluble ions (both anions and cations) was analyzed using ion chromatography (IC) on a Dionex 5000 instrument (Dionex Co., Sunnyvale, CA, USA). The elemental contents in the other part of the same substrates were analyzed using scanning electron microscopy/energy-dispersive X-ray spectroscopy (SEM/EDS) techniques. A scanning electron microscope (Indusem, Tescan Orsay Holding a.s., Brno, Czech Republic), equipped with energy-dispersive X-ray spectroscopy (XFlash detector 5010, Bruker, Karlsruhe, Germany) was used for this purpose. The relative mass shares of elements analyzed by SEM/EDS were converted to concentrations in µg/m^3^, based on the concentrations of sulfates from the ion chromatography and the relative share of sulfur in a sample from the EDS, under the assumption that all sulfur is soluble and present in the form of sulfates. The detection limit for the elements was 0.3 atomic%.

### 2.4. Environmental Contamination

Values of the air levels of SO_2_, O_3,_ nitrogen oxides (NO_2_), and particulate matter (PM)_2.5_ and PM_10_, available from the National Hydrometeorological monitoring system and recorded every hour, were sourced from the nearest stationary monitoring station. The research plant with the ambulatory room for the subjects’ examination was located at the periphery of the city, approximately 3.0 km from the hydrometeorological monitoring station.

### 2.5. Collection of Biological Samples and Analysis of Oxidative Stress Biomarkers

As in our previous study, an EcoScreen Turbo DECCS device (Jaeger, Germany) was used for the collection of EBC [15], under the same conditions as in 2016. Exhaled air volume and EBC volume during 15 min of breathing were recorded for every subject. In addition, plasma and urine samples were collected. All samples were spiked with deuterium-labeled internal standards and stored at −80 °C prior to analysis.

A set of eight selected oxidative stress biomarkers was analyzed after solid-phase extraction (SPE) using liquid chromatography-electrospray ionization-tandem spectrometry (LC-ESI-MS/MS), as described by Syslova, Kacer, Neprasova and coworkers [20,21,22,23]. Laboratory personnel were blinded to sample codes.

The limit of detection (LOD) was 1.2 ± 0.2 ng/mL for MDA and C6-12, and 1.2 ± 0.2 pg/mL for 8-isoprostane, 8-OHdG, 8-OHG, 5-OHMeU, o-Tyr and 3-NOTyr. The standard error was 3.0%. Biomarker levels in urine samples were normalized to urinary creatinine concentration, according to the method of Jaffe [24].

### 2.6. Statistics

The data are presented as mean ± standard deviation (SD). An independent group t-test was performed to assess differences between exposed individuals and control subjects. A paired *t*-test was used to compare repeated measurements in groups of exposed and control subjects within each year (pre-shift vs. post-shift and morning vs. afternoon samples). Bivariate associations between studied variables were assessed by Pearson’s product-moment correlation coefficient. Statistical significance was tested at the significance level α = 0.05 level. SPSS statistical software version 25.0 (SPSS, Chicago, IL, USA) was used for statistical analyses.

## 3. Results

### 3.1. Subjects

General characteristics of the groups of workers and control subjects over all three years are shown in Table 1. There were no statistically significant differences in the number of participants, age, gender, BMI, smoking (including packyears in smokers and ex-smokers) and alcohol consumption between the two groups studied in any year (all *p* > 0.05).

As to the symptoms, no difference between exposed and control subjects was found in the prevalence of dyspnea, cough or common cold symptoms (all *p* > 0.05).

There was no difference between the workers and control subjects in the proportion of those who had breakfast in the morning and of those who smoked in the morning, before the pre-shift examination. Similarly, the proportion of those who had lunch and those who smoked before the post-shift or afternoon examination was not significantly different.

#### 3.1.1. Exposure Characteristics

Exposure data of the groups of workers are presented in Table 2. As can be seen, the mean total exposure in 2016 was 18 ± 10 years, which decreased to 12.2 ± 9.3 years in 2017, as several of the workers retired. This difference, however, did not reach significance (*p* = 0.071). The usual daily exposure and latency since the last exposure in the years 2016–2018 did not differ.

The monitoring days’ exposure was organized to be longer than the typical average length in order to cover at least the mean ± standard deviation of the group studied.

As in the previous study in 2016, no personal respiratory protection was used in the workshops during any of the operations. The workers only used goggles for all working operations and a welding shield for the welding operations.

#### 3.1.2. Spirometry

There were no differences in 2017 and 2018 between exposed workers and control subjects in lung function parameters, and no post-shift decrease in these parameters was found in both years of follow-up (data not shown).

### 3.2. Workplace Aerosol

#### 3.2.1. Mass Concentrations and Mass Share of Analyzed Elements

Total mass concentration in the workshops obtained from BLPI was 0.351 mg/m^3^ during machining in 2017; and 0.179 and 0.217 mg/m^3^ during machining and welding, respectively, in 2018. Aerosol mass concentrations were variable during the monitoring days; however, the mass size distributions were always monomodal for the welding process and bimodal for machining. Two modes recorded at machining were probably caused by two different processes: the large particles were released mechanically as the machining tool collided with material irregularities of its surface layer; the ultrafine mode was formed by condensation of compounds previously evaporated from the heated surface of the ground materials.

Overall, mass concentrations determined by gravimetric analysis were higher in 2016 in comparison with both 2017 and 2018; however, the mass share of analyzed elements in nanoparticle size fractions within the first two stages of the cascade impactor (<100 nm) was higher for machining in 2017 and 2018, than for this operation in 2016. It is shown in Table 3. Importantly, the mass share of analyzed elements is minor in relation to the total mass of each sample, where most of the mass is formed by inorganic salts and organic matter, including those coming from outdoors. On the other hand, the elements subsequently analyzed by SEM/EDS in the nanoparticle size range were mostly emitted from the analyzed operations, and the sources were the samples and the machining tools.

#### 3.2.2. Number Concentrations

The results of measurements in the workshops during 2016–2018 are presented in Table 4, Table 5 and Table 6. The data in Table 4 shows the median number concentrations of four particulate matter (PM) fractions (<100 nm; 100 nm–1 µm; 1–10 µm; total <10 µm), measured by online monitoring instruments (SMPS and APS) during the shift at individual working operations, and, in the background, measured overnight prior to the start of the individual working process in the workshop.

Proportions of these fractions during the shift related to the specific working processes are presented in Table 5. In 2016 and 2018, when welding was included in the study, the proportion of nanoparticles observed was always lower than at machining. The varying proportion of nanoparticles on the background is mostly given by varying ambient conditions.

Detailed results, including the background measurements, are available in Figures S1 and S2, where the time evolution of particle number concentrations during the 2017 and 2018 studies can be seen. Four size fractions are shown separately (sum of particle number concentrations for <100 nm, 100 nm–1 µm, 1–10 µm and total <10 µm). Each of the two backgrounds can be characterized by relatively stable concentrations in all recorded fractions. It confirms that there were no indoor sources emitting aerosol particles. At machining, the concentrations in all fractions also remained stable because machining operations emitted steady amounts of particles in time. On the other hand, the time evolution of particle number concentrations at welding reflects the quasi-periodic character of the process (shown by the fluctuations of submicrometer particle concentrations).

These data also confirm that, despite the changing processes in investigations of the exposed researchers, the proportion of nanosized particles remained relatively high over all three years.

#### 3.2.3. Elemental Composition

The percentage of elements detected by SEM/EDS smaller than 100 nm can be seen in Table 6. Mg, Ti and P were also measured; however, their proportion was below their detection limit (approximately 0.1, 0.3 and 0.2 weight%, respectively).

Fe was the most commonly occurring element during welding, smelting (in 2016) and machining. The electrodes used in welding were the main origin of F^−^. Geopolymers were the source of Si. On the other hand, this analysis did not detect organic compounds, amines and water-soluble compounds.

If we focus on the only operation carried out in all three consequent years, which is machining, shown in Table 3, we can observe a clearly increasing trend in the sums of elemental concentrations determined by SEM/EDS in the nanometer size range from 2016, through 2017 and 2018. We can assume that these elements were emitted mostly indoors during the operations.

### 3.3. Oxidative Stress Markers

#### 3.3.1. EBC

**Figure 1:**
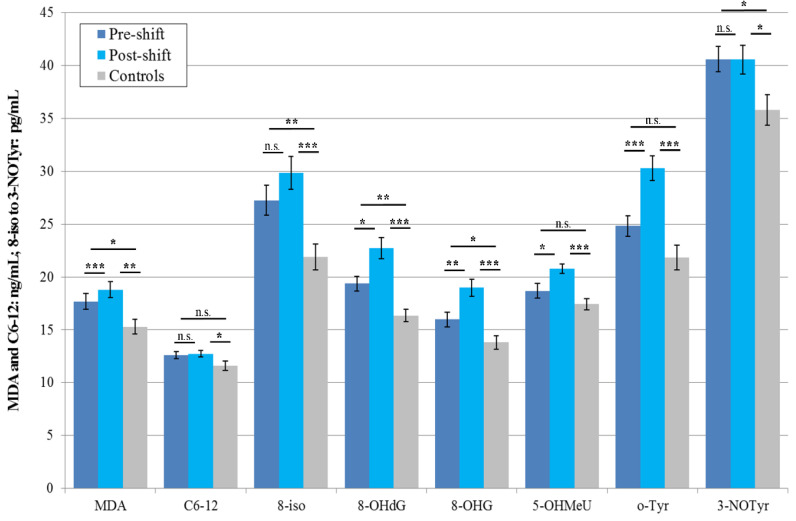
Mean and standard deviations of the levels of eight oxidative stress markers in the EBC of 20 workers (machining) and 20 control subjects (morning) in 2017, * *p* < 0.05, ** *p* < 0.01, *** *p* < 0.001, n.s. = not significantly different, EBC = exhaled breath condensate, MDA = malondialdehyde, C6-12 = aldehydes C6-12, 8-iso = 8-*iso*-prostaglandin F2α (8-isoprostane), 8-OHdG = 8-hydroxy-2-deoxyguanosine, 8-OHG = 8-hydroxyguanosine, 5-OHMeU = 5-hydroxymethyl uracil, o-Tyr = *o*-tyrosine, 3-NOTyr = 3-nitrotyrosine.

In accordance with the results in 2016 (when seven of eight monitored markers were elevated), the concentrations of five pre-shift markers of the workers in 2017 were higher in the EBC than in control subjects, and five were increased post-shift, compared with pre-shift values. All workers during this monitoring shift performed machining. All eight post-shift markers of the exposed subjects were higher, as compared with the control subjects, as can be seen in Figure 1.

In 2018, three pre-shift markers’ concentrations for the workers who were chronically exposed to nanoparticles were higher than in the morning samples (C6-12, 8-isoprostane, and 8-OHdG) of the control subjects. In this year (2018), when exposure included two main operations (machining and welding), the levels of all eight markers increased in the workers post-shift, compared with pre-shift levels.

**Figure 2:**
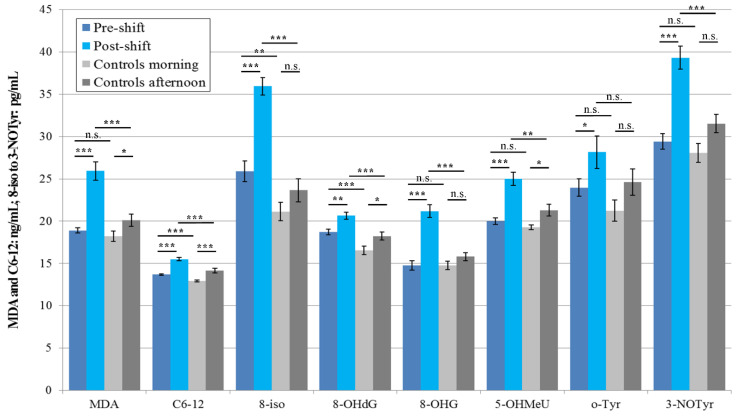
Mean and standard deviation of the levels of eight markers of oxidative stress in the EBC of the 21 workers (shift = welding or machining) and 18 control subjects in 2018, * *p* < 0.05, ** *p* < 0.01, *** *p* < 0.001, n.s. = not significantly different, EBC = exhaled breath condensate, MDA = malondialdehyde, C6-12 = aldehydes C6-12, 8-iso = 8-*iso*-prostaglandin F2α (8-isoprostane), 8-OHdG = 8-hydroxy-2-deoxyguanosine, 8-OHG = 8-hydroxyguanosine, 5-OHMeU = 5-hydroxymethyl uracil, o-Tyr = *o*-tyrosine, 3-NOTyr = 3-nitrotyrosine.

Rather surprisingly, in this year (2018), when both groups studied were examined twice, the levels of the four afternoon markers increased in the control subjects as compared with the morning samples (MDA, C6-12, 8-OHdG, and 5-OHMeU). However, the levels of all these elevated afternoon markers were still significantly lower than the levels of post-shift markers in the workers. The results are shown in Figure 2.

#### 3.3.2. Plasma

**Figure 3:**
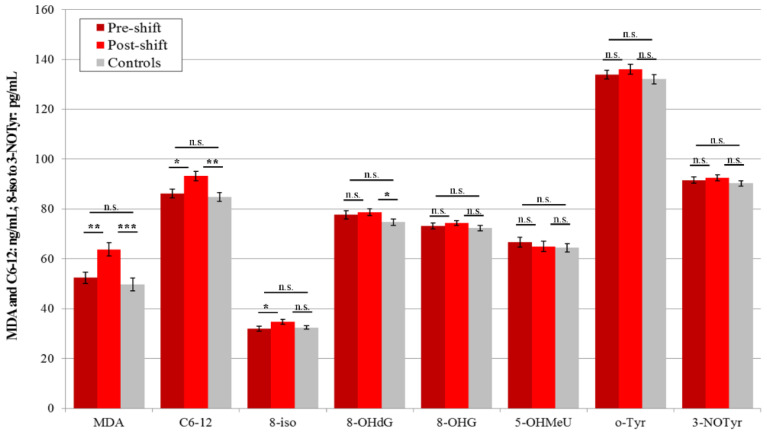
Mean and standard deviation of eight oxidative stress markers in the plasma of 20 workers (shift = machining) and 20 control subjects (morning) in 2017, * *p* < 0.05, ** *p* < 0.01, *** *p* < 0.001, n.s. = not significantly different, MDA = malondialdehyde, C6-12 = aldehydes C6-C12, 8-iso = 8-*iso*-prostaglandin F2α (8-isoprostane), 8-OHdG = 8-hydroxy-2-deoxyguanosine, 8-OHG = 8-hydroxyguanosine, 5-OHMeU = 5-hydroxymethyl uracil, o-Tyr = *o*-tyrosine, 3-NOTyr = 3-nitrotyrosine.

In 2017, the absolute values of the levels of seven pre-shift markers in plasma of the workers were higher than in the control subjects (except 8-isoprostane), but significance was not reached, and the concentrations of three markers (MDA, C6-12, and 8-isoprostane) increased post-shift vs. pre-shift in the workers who all performed machining. In addition, three post-shift markers’ levels (MDA, C6-12, and 8-OHdG) were significantly higher in the workers compared with the samples of the control subjects. The results are presented in Figure 3.

In 2018, the levels of six pre-shift plasma markers (MDA, C6-12, 8-isoprostane, 8-OHG, 5-OHMeU, and o-Tyr) were significantly higher in the workers, as compared to morning samples of the control subjects.

**Figure 4:**
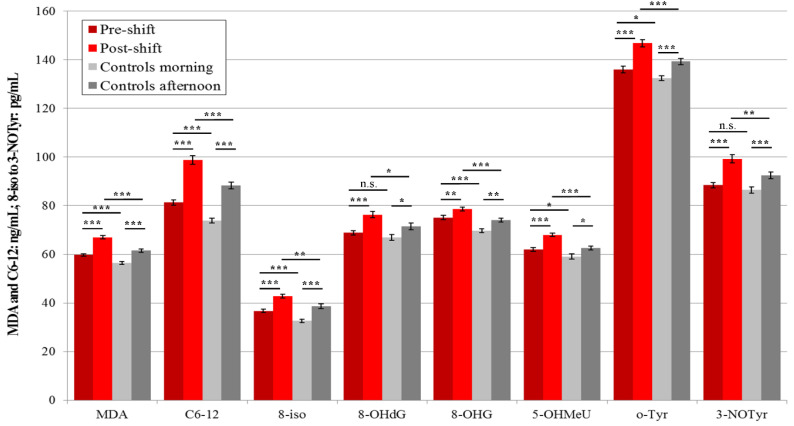
Mean and standard deviation of the levels of eight oxidative stress markers in the plasma of 21 workers (shift = welding or machining) and 18 control subjects in 2018, * *p* < 0.05, ** *p* < 0.01, *** *p* < 0.001, n.s. = not significantly different, MDA = malondialdehyde, C6-12 = aldehydes C6-C12, 8-iso = 8-*iso*-prostaglandin F2α (8-isoprostane), 8-OHdG = 8-hydroxy-2-deoxyguanosine, 8-OHG = 8-hydroxyguanosine, 5-OHMeU = 5-hydroxymethyl uracil, o-Tyr = *o*-tyrosine, 3-NOTyr = 3-nitrotyrosine.

The concentrations of all eight markers in the second samples (i.e., post-shift, after machining and welding) for both groups of subjects, including all afternoon samples of the control subjects, significantly increased. 100% of the post-shift marker concentration levels in the workers were significantly higher compared to the afternoon samples of the control subjects, as shown in Figure 4.

#### 3.3.3. Urine

**Figure 5:**
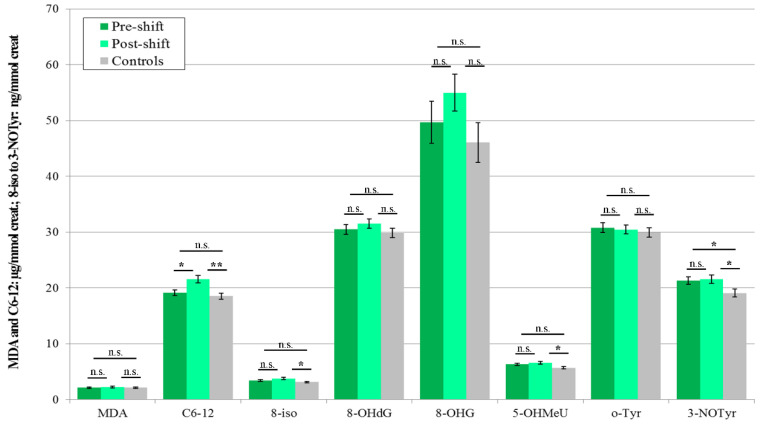
Mean and standard deviation of the levels of eight oxidative stress markers in the urine of 20 workers (shift = machining) and 20 control subjects (morning) in 2017, * *p* < 0.05, ** *p* < 0.01, n.s. = not significantly different, creat. = creatinine, MDA = malondialdehyde, C6-12 = aldehydes C6-C12, 8-iso = 8-*iso*-prostaglandin F2α (8-isoprostane), 8-OHdG = 8-hydroxy-2-deoxyguanosine, 8-OHG = 8-hydroxyguanosine, 5-OHMeU = 5-hydroxymethyl uracil, o-Tyr = *o*-tyrosine, 3-NOTyr = 3-nitrotyrosine.

Urine samples have shown less significant differences within the groups studied. In 2017, only the level of one marker (3-NOTyr) was elevated in the worker pre-shift vs. control subjects, and only one increased in the workers post-shift (C6-12). However, the concentrations of four markers were higher in the post-shift samples (C6-12, 8-isoprostane, 5-OHMeU, and 3-NOTyr) compared with the control subjects. The results are shown in Figure 5.

**Figure 6:**
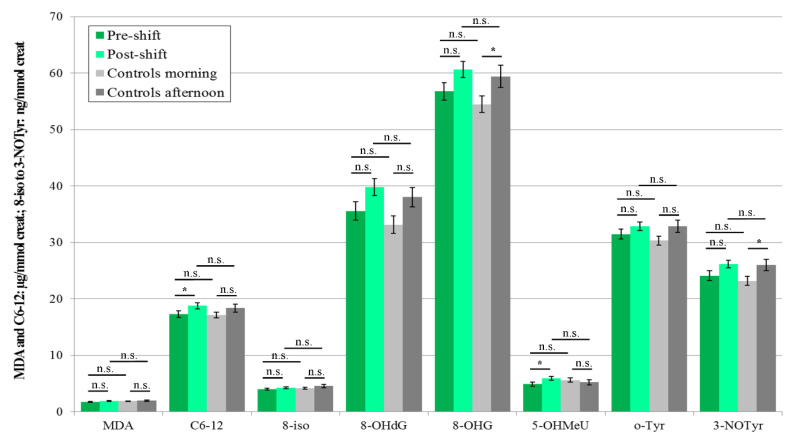
Mean and standard deviation of the levels of eight oxidative stress markers in the urine of 21 workers (shift = welding or machining) and 18 control subjects in 2018, * *p* < 0.05, n.s. = not significantly different, creat. = creatinine, MDA = malondialdehyde, C6-12 = aldehydes C6-C12, 8-iso = 8-*iso*-prostaglandin F2α (8-isoprostane), 8-OHdG = 8-hydroxy-2-deoxyguanosine, 8-OHG = 8-hydroxyguanosine, 5-OHMeU = 5-hydroxymethyl uracil, o-Tyr = *o*-tyrosine, 3-NOTyr = 3-nitrotyrosine.

In 2018, the majority of markers in the workers had higher absolute levels, both in the pre-shift vs. morning samples in the control subjects and post-shift vs. afternoon samples of the control subjects, although the difference was not significant, as shown in Figure 6. The concentrations of two markers (C6-12 and 5-OHMeU) increased in the workers post-shift, and two (8-OHdG and 3-NOTyr) in the control subjects in the afternoon vs. morning samples.

### 3.4. Acute Exposure Effect

Significant elevations of the post-shift vs. pre-shift markers in 2017 are shown in Figure 1, Figure 3 and Figure 5, and those from 2018 in Figure 2, Figure 4 and Figure 6. The shift effect was most pronounced in EBC (81.3%), less in plasma (68.8%), and least in the urine samples (18.8%).

When the years 2017 and 2018 are compared, the concentrations of nine (37.5%) total markers from all fluids increased in 2017, when all workers carried out machining, and 17 (70.1%) markers in 2018, when they were divided into the welding and machining process.

### 3.5. Chronic Effect

#### 3.5.1. Three Years’ Analysis of EBC Samples

**Figure 7:**
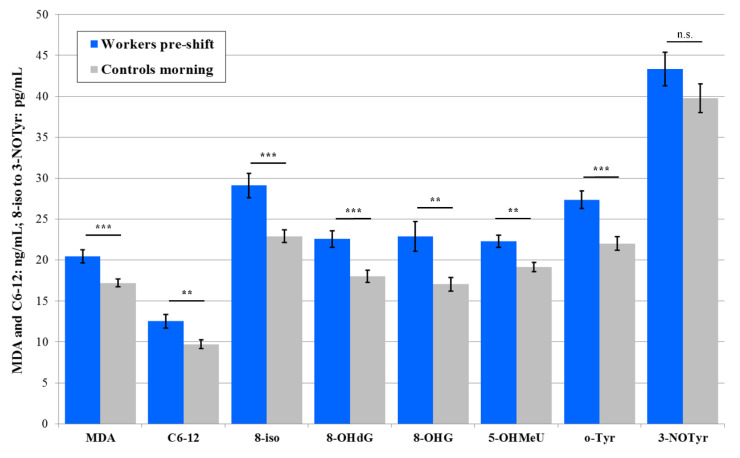
Mean and standard deviation of the levels of eight markers of oxidative stress in the EBC of all 29 workers pre-shift and in all 39 control subjects in 2016, 2017 and 2018; each person is included once only (a mean of repeated marker values), ** *p* < 0.01, *** *p* < 0.001, n.s. = not significantly different, EBC = exhaled breath condensate, MDA = malondialdehyde, C6-12 = aldehydes C6-C12, 8-iso = 8-*iso*-prostaglandin F2α (8-isoprostane), 8-OHdG = 8-hydroxy-2-deoxyguanosine, 8-OHG = 8-hydroxyguanosine, 5-OHMeU = 5-hydroxymethyl uracil, o-Tyr = *o*-tyrosine, 3-NOTyr = 3-nitrotyrosine.

Aggregated data from all three years for workers from the pre-shift samples and control subjects’ morning samples are shown in Figure 7. For each subject who had repeated examinations, a mean of those values of EBC markers was calculated. A total of seven markers (87.5%; with the exception of 3-NOTyr) showed a significant difference between the two groups of subjects, showing a chronic effect.

#### 3.5.2. Trends in 17 Repeated Examinations in 3 Biological Fluids

To evaluate the trend of chronic effects, an analysis of the pre-shift oxidative stress markers in all samples of 17 workers participating in both years 2017 and 2018 was carried out. The results showed that the levels of five total markers (20.8%) in the three biological fluids were elevated, and the levels of three markers (12.5%) decreased. Their occurrence, however, was inconsistent throughout the years.

#### 3.5.3. Occupational Factors

- Years of exposure

In 2017 only, a correlation of the duration of employment in years with post-shift EBC 8-OHG level (0.611; *p* = 0.007) was observed.

In 2018, no positive correlation of the duration of employment in years with any concentration of markers in the pre-shift EBC was observed.

- Daily exposure length

In 2017, positive correlations with pre-shift levels of EBC o-Tyr (0.538; *p* = 0.015) and plasma C6-12 (0.461; *p* = 0.041) were observed. In urine levels, the correlation of o-Tyr (0.607; *p* = 0.005) and 3-NOTyr (0.649; *p* = 0.037) with the duration of daily exposure was observed. However, the same correlations were not observed in 2018, nor any others.

- Latency since last exposure and pre-shift markers

In 2017, a negative correlation with the pre-shift plasma concentration of 8-isoprostane (−0.460; *p* = 0.042) with the latency since last exposure was observed.

### 3.6. Individual Characteristics

Twenty parameters and questions in the questionnaire related the age, gender, alcohol intake (occasionally/never), smoking (yes/no), regular physical activity (more than 30 min daily), medication, diseases, symptoms, last meals ingested, and recent smoking.

#### 3.6.1. Workers Pre-Shift in 2017 and 2018

The correlations of individual characteristics with pre-shift markers were investigated and compared in order to study chronic effects. The numbers were relatively low, as among the total 20 parameters analyzed and eight markers, for each biological fluid, there were a total of 19 positive correlations (1.38%) and 28 negative correlations (2.32%) in both years. The highest number of positive correlations in the pre-shift samples was in the urine (1.56%), and the lowest in the EBC (0.31%). However, no consistency was observed between these two years (2017 and 2018), as the markers did not repeat in the other year. The only exception was for hypothyroidism treated with thyroxine, which showed a positive correlation for 8-OHG in the pre-shift plasma in 2017 (*p* = 0.045) and in 2018 (*p* = 0.012).

#### 3.6.2. Workers Post-Shift in 2017 and 2018

The same individual characteristics were studied with the post-shift markers in both years in order to evaluate the acute effect of exposure, with a total of 30 correlations seen (11 positives (1.15%) and 19 negatives (1.38%)). The highest amount of positive correlations with the individual parameters was observed in the EBC (0.52%). Plasma and urine both showed a lower amount of positive correlations (0.31%).

The only marker present in both years was 3-NOTyr in the post-shift EBC, which was positively correlated with occasional ethanol ingestion both in 2017 (*p* = 0.028) and in 2018 (*p* = 0.041).

#### 3.6.3. Control Subjects in 2018

In the control subjects in 2018, among correlations of the 20 particular non-occupational parameters with the oxidative stress markers in the morning samples, a total of 5 markers correlated positively (1.04%) and 11 negatively (2.29%); however, in the afternoon samples, the number of positive correlations increased to 13 (2.71%), and 10 correlated negatively (2.08%). In total, there were 1.88% positive and 2.19% negative correlations. The majority of positive correlations were observed in the EBC (2.29%) and the least in the plasma (0.33%). Again, a correlation of the markers with these parameters was never observed both in the morning and in the afternoon.

From the 13 positively correlated afternoon markers, 4 in the EBC correlated with cough (MDA, 8-OHdG, 5-OHMeU and 3-NOTyr, all *p* < 0.05), two EBC markers with smoking (C6-12 and 5-OHMeU), one with a feeling of a common cold (C6-12), and one with taking any medication (MDA). One plasma marker correlated with smoking (5-OHMeU), one with hypertension (3-NOTyr), and one with body mass index (BMI) (8-isoprostane). Two urine markers correlated with occasional alcohol intake (C6-12, 8-OHdG); however, these correlations were not seen in the morning samples.

### 3.7. Correlation of One Marker in Different Fluid Samples

The identical pre-shift marker positively correlated a total of nine times with the level of the same marker in another body fluid. Four times, it was positively correlated with the same body fluid post-shift marker (in 2017 for pre-shift MDA, C6-12, 8-isoprostane, and o-Tyr) in the post-shift EBC. Further, two positive correlations were seen between urine (MDA) and plasma (C6-12) samples taken at the same time. In addition, two negative correlations were seen. These correlations involved only the workers and were rare.

### 3.8. Environmental Contamination

The levels documented from the national hydrometeorological monitoring system did not exceed the allowed limits and were classified as low or very low during all measurements. No consistent correlations with oxidative stress markers in 2017 and 2018 were observed.

## 4. Discussion

It has been repeatedly declared that there is an urgent need to find appropriate biomarkers to control the level of oxidative stress in the most-exposed human subjects in order to prevent potentially severe consequences [25,26,27]. However, data on the markers of oxidative stress in the literature are scarce.

Our first study describing the data from this research plant in 2016 has proven that occupational exposure was the primary cause of the elevation in the concentration of the biomarkers of oxidative stress in the EBC, both pre-shift, related to the chronic exposure, and post-shift, related to the impact of the exposure during the monitoring day.

The new results presented here, for both pre-shift and post-shift measurements, showed a similar trend in both follow-up years in all biological fluids, even if significance has not been reached for some markers. This may be related to the limited number of subjects available for examination.

This study examined the control subjects in 2018 twice, at two different times, and found new, rather unexpected results in the afternoon samples, where the levels of 50% of total markers increased. Despite this elevation, the results for the workers in 2018 were significantly higher in 100% of these findings, compared to those of the control subjects. The findings led us to analyze a set of 20 non-occupational parameters and environmental contamination data in order to explain this observation.

- Acute effect

The level of workplace aerosol exposure measured by objective methods was relatively high in all three years, as was the proportion of particles under 100 nm.

In this study, we attempted to compare the effect of acute exposure of workers in different working processes, where, for all three years, only EBC results were available. In 2016, only 37.5% of EBC markers were elevated post-shift when the shift included three operations: welding and smelting in workshop 1 and machining in workshop 2. In 2017, the proportion of elevated markers increased to 62.5%, when all workers performed machining. In the final year (2018), exposure consisted of welding in workshop 1 and machining in workshop 2, and the elevation was observed for 100% of EBC samples. MDA appeared to be the most responsive marker, as it elevated in the post-shift EBC every year.

If we compare the aerosol exposure in these three years, we find that the highest exposure to all nanoparticles during the shift was observed in 2016, significantly lower in 2017 and again lower in 2018, both when we use mass concentrations and when we use number concentrations (Table 4) as the exposure metrics. This observation goes against the trend in biomarkers concentration found in EBC in the years 2017 and 2018 (Figure 1 and Figure 2).

On the other hand, if we compare the mass concentrations summed up in all of the years by SEM/EDS analyzed elements, determined at machining—the only working operation used in all three years—the exposure increased between the years 2016, 2017 and 2018, respectively, in the ratio of 1, 4:5, 5:8 (as shown in Table 3), respectively, in accordance with the increasing trend in biomarkers found in EBC in the last two years. As already mentioned, these elements were largely emitted by machining operations with origin from the samples and machines. On the other side, the total mass concentrations, determined by gravimetry on the same samples, contained many inorganic salts and organic material, including those coming from outdoors.

Accordingly, in another study by Ricelli et al., EBC levels of MDA were elevated post-8 h occupational exposure to stainless steel tungsten inert gas welding fumes, which contained Cr and Ni. In addition, the EBC Cr concentration was higher post-shift (0.08 μg/L) than pre-shift (0.06 μg/L), confirming the absorption of Cr due to inhalation exposure [28].

In a study of fire-fighters by Wu et al., there was a marginal increase of 8-isoprostane levels in the post-shift EBC samples on burn days (3.5–3.8 pg/mL; *p* = 0.06) compared to non-burn days (2.86–3.20 pg/mL) [29]. These post-shift levels were approximately 10-fold lower than the concentrations in this study’s worker subjects post-shift, as a less sensitive method, the enzyme-linked immunosorbent assays (ELISA), was used in that study.

- Chronic effect

The chronic effect in our workers, evaluated by the pre-shift EBC samples, slowly decreased, from 87.5% in 2016 to 62.5% in 2017, and to 37.5% in 2018. Among the eight markers examined, only 8-OHdG concentration was elevated pre-shift in all three years. As the usual daily exposure time reported by the workers has not shortened, this may be explained by the fact that the total average duration of exposure in years was lower in 2017 and 2018. However, this difference did not reach significance. We also speculate that the workers may have behaved more cautiously, as a reflection of the results of our study from 2016. Anyway, the personnel respiratory protective equipment has not been used in any year.

Similarly, in the above-mentioned occupational study focused on nanoparticles, a chronic/subacute effect on the EBC MDA concentrations was observed due to exposures to tungsten inert welding fumes. The pre-shift MDA levels were higher on Friday, i.e., at the end of the week, compared to the pre-shift sample on Monday [28].

- Duration of exposure and latency

The length of exposure, and the time lag from the end of exposure to sample collection, may be important in the evaluation of post-exposure samples. In our recent study, the samples were collected within 1 h after 2.5–3.5 h exposure; however, other studies show that the levels of the markers can further rise, becoming even more significantly different.

In the study by Graczyk et al., which measured 8-OHdG levels in the plasma and urine, after 60 min lasting exposure to tungsten inert gas welding fumes, marker elevations were not seen 1 h post-exposure but appeared at 3 h post-exposure. These authors recommended analysis at even later time points of 12 h and 24 h post-exposure [30].

In the report by Riccelli et al., the EBC collections after 8 h shift exposure showed an increased level of MDA [28]. Similarly, our earlier studies found the elevation in EBC markers concentrations for the workers exposed to metal oxide nanoparticles who worked 8 h shifts (samples were collected immediately following shifts) [31,32,33]. In both EBC and urine (but not plasma, due to a noninvasive approach being desired), markers of oxidative stress were measured in nano-Fe-oxides exposed workers. Despite observing an increase in the levels of the EBC markers, the difference in the urine markers concentrations was less significant. Urine markers, however, showed the same trend, and some correlated with the EBC levels for markers such as MDA and C6-C12 [21]. Similarly, the nano-TiO_2_ exposed workers with higher exposure also had elevated urine markers concentrations [32]. It is plausible that the elevation of urine markers levels may be dose-related, as these were not elevated in the subjects with significantly lower exposure, such as office employees of nano-TiO_2_ manufacturing plants, despite the significantly higher concentrations of markers of oxidation of nucleic acids, proteins [34] and lipids in the EBC [35].

The patients with silicosis and asbestos-induced diseases, with carcinogenic silica or asbestos persisting in their lungs, displayed elevated EBC markers and plasma and/or urine markers levels, even decades after their exposure ceased [36]. The mechanism of oxidative stress is the key point and may be a concern regarding nanoparticles’ exposure.

The type and route of exposure are also important. The elevation of oxidative stress markers in the EBC, plasma and urine after UV irradiation in the solarium was observed after just 3 h. The skin irradiation of 80% of the body also resulted in a pronounced systemic effect [37].

- Workshops

The regular working operations of the workers in the workshops were identical throughout all years. On the other hand, the operations selected for the monitoring day changed, which may have contributed to the differences related to individual markers. In addition, over the years, slightly different samples were selected for machining on the monitoring day, based on how the scientific interests of researchers have changed. This probably also affected the proportion of the analyzed elements in the working atmosphere. Obviously, not only the quantity and proportion of nanoparticles but the chemical composition of the aerosol may also play a role, especially Fe [31].

When we merged the results of the biomarkers in workers performing welding in 2016 and 2018 and compared the levels with the results of the workers who performed machining, the results did not differ significantly in any of the eight monitored markers of oxidative stress. This may mean that higher numbers of subjects are needed to find differences. A difference was noticed in our pilot cytogenetic study; specifically, an increase of chromosomal breaks in a subgroup of researchers performing welding and smelting in 2016 [38]. Therefore, in our recent research project (No. GACR 18-02079S of the Czech Science Foundation), we continue the follow-up of the workers to evaluate potential genetic and epigenetic changes [39].

- Methodology

The accuracy of the EBC collection and analysis has been repeatedly questioned with regard to its reliability, as there is no marker available to assess its dilution that is as adequate as creatinine in urine [15]. However, regardless of the absence of such a marker, an identical trend can be seen across Figure 1, Figure 2, Figure 3, Figure 4, Figure 5 and Figure 6 (i.e., in the levels of both pre-shift and post-shift markers in the workers in all fluids examined, compared with the control subjects). These trends confirm the value of EBC marker analysis and are consistent with our earlier studies.

A systematic review by Shoman et al. and meta-analysis of EBC 8-isoprostane by immunological methods resulted in the determination of a reference level, with geometric mean 7.67 pg/mL (95% confidence interval 5.58–9.76) [1], which is lower compared with our average value of 21 pg/mL in the control subjects and 26 pg/mL in the workers pre-shift.

The healthy adults had 8-OHdG levels 3.9 ng/mg creatinine (interquartile range 3–5.5 ng/mg creatinine) in the urine according to a meta-analysis of the studies by Graille et al. and LC-ESI-MS/MS analysis was superior to immunological methods, due to its higher sensitivity and specificity [3]. Our control subjects had an average level of 8-OHdG, approximately 0.3 ng/mg creatinine, and no results were below the LOD [30].

Unfortunately, results from human studies focusing on the markers of oxidation of lipids, nucleic acids and proteins show great variability. They are additionally complicated by the expression of results in different gravimetric and molar units. This occurs especially for urine samples, where several combinations of units may be used. For better comparison, we present the results of our analyses in EBC, plasma and urine in the groups of control subjects and workers in different variants of gravimetric and molar units in Tables S1–S6.

- Age, gender, BMI

A detailed analysis of potential correlations of the markers of oxidation with individual factors related to personal history, diseases, all types of medication, habits, and lifestyle was carried out.

As shown in Table 1, the age of the workers throughout the years 2016–2018 did not differ. No correlation with age was seen in the markers in all fluids studied, similar to our earlier studies in exposed workers. Accordingly, in the EBC, no influence of age, gender and BMI was found by Shoman et al. in a recent meta-analysis of studies investigating 8-isoprostane, a marker of oxidation of lipids in the EBC [1].

For the plasma marker levels, however, some studies show unequivocal results concerning the influence of age and/or gender. In the recent study by Pinchuk et al., 3-NOTyr was independent of these parameters; however, MDA was lower in the women aged 50–55 years compared with men, which may relate to a change of metabolism post-menopause [5]. In the study by Weber et al., plasma 3-NOTyr correlated with subject age in the range of 55–75 years [40]. These authors proposed the existence of different types of oxidative stress, some of which may depend on hormone levels.

For the markers in the urine, limited data are available, showing that similarly to our study, 8-OHdG in the urine was not gender-related [3]. Interestingly, urine MDA levels were lower in the descendants from long-living families, theorized to be better genetically equipped to handle oxidative stress [40]. In our study, these factors did not interfere with each other, as there were no women in the postmenopausal age and only one male above 65 years of age who participated in the control subject group in 2018.

Experimental data indicate that obesity is associated with increased oxidative stress, and data from human studies have shown that weight loss in obese patients leads to a decrease in markers of oxidative stress in white blood cells. These markers in urine still displayed a high variety of oxidized nucleic acids [41]. In our study, the groups did not differ in BMI, and no correlation was observed.

- Smoking, alcohol

The increased effect of smoking on the biomarkers has been found in several studies, as documented in a meta-analysis focusing on 8-OHdG and its increase in the urine [3]. On the other hand, smoking did not affect 8-isoprostane levels in EBC [1]. In our study, there was no difference in markers between groups of subjects for any bodily fluid studied, and the proportion of smokers was low. There was a minority of abstainers from alcohol in the groups studied. However, no consistent correlations between the levels of the markers in any fluid have been observed for smoking and alcohol intake.

- Food

A review of the studies by Brieger et al. has concluded that diet and drinking of non-alcoholic beverages may impact the concentration of oxidative stress markers and contribute to their variability [42]. The effect may depend on the time interval of the sampling [15]. For the volunteers, consumption of a median size meal (8.5 kcal/kg of body weight) increased 8-isoprostane levels in the plasma at 3 h and 6 h post-meal; however, levels did not increase in the EBC in the study by Kurti et al. [43]. Drinking 1 L of Coke (pH 2.5) or mineral water (pH 6.5) decreased pH in the EBC, blood and urine of the volunteers within 15 min [44].

In a controlled study by Kanabrocki et al. in 12 male, healthy volunteers, with a daily distribution of calories by a meal of 29% for breakfast, 30% for lunch and 41% for dinner, peak levels of urine 8-OHdG, MDA, and 8-isoprostane concentrations occurred in the evening, which may be attributable to the ingestion of a greater number of calories during dinner [45]. In the workers and control subjects in this study, no difference in the concentration of these markers was observed if breakfast was consumed before the pre-shift/morning sampling, nor if their last meal was the previous evening’s dinner. Before and during EBC sampling, only tap water was provided for regular intake.

- Physical activity

Habitual level of physical activity may impact 8-isoprostane production [46], and its elevation in the EBC was found in a study by Kurti et al. after exhaustive exercise for postmenopausal women [47]. The effect of intense work on MDA, another oxidative stress marker, is more complex. It may depend on the fitness of the subject and correlate inversely with the initial level of the given biomarker prior to exercise [48].

- Symptoms

In our previous study in 2016, dyspnea and chronic bronchitis in the exposed subjects were more frequent and correlated with pre-shift o-Tyr and post-shift 3-NOTyr levels in the EBC. In both years of follow-up, only one worker had a chronic cough, who had 18 years’ accumulated exposure to nanomaterials in 2017 and did not participate in 2018. Among the control subjects in 2018, one subject was accidentally included who complained of a cough first in the afternoon. This control subject had 3-NOTyr, MDA, 8-OHdG, and 5-OHMeU positively correlating with a cough, associated with newly appearing common cold symptoms. In the literature, the 3-NOTyr level was increased in patients with severe asthma, similarly to 8-isoprostane and further oxidative stress and inflammation EBC markers, such as leukotrienes C4–E4 [49,50].

In spirometry findings, a significant decrease in post-shift levels of % FEV1 and FEV1/FVC in the workers was observed in 2016. This was not observed in this follow-up study, which may be explained by the marginally shorter total length of exposure during the years 2017 and 2018.

- Diseases

Oxidative stress has been associated with aging, diabetes, cancer, neurodegenerative, among other disorders [50]. In this study, the level of oxidative stress marker 8-OHG correlated with hypothyroidism treated with thyroxine. This disease was present in two female workers in 2017 and 2018, and in two female control subjects in 2018, as the only disease with a positive correlation with pre-shift 8-OHG plasma level in the workers. However, this correlation was not observed in the control subject samples in 2018. The relationship with oxidative stress is plausible; as for thyroid function, attaching an iodide to thyroglobulin requires hydrogen peroxide [42]. However, no subject displayed typical hyper- or hypothyroidism symptoms.

- Circadian fluctuations

A link between the molecular mechanisms of circadian rhythms and oxidative stress has been established, but it is still insufficiently understood. Importantly, oxidative stress biomarkers display considerable variation in healthy populations, and diurnal variability may vary for different biomarkers measured in different bodily fluids [51]. For example, afternoon levels for plasma isoprostanes were lower in comparison with the morning samples [52]. On the other hand, no significant circadian fluctuations of EBC, plasma and urinary MDA and 8-OHdG concentrations were seen in healthy young men at sampling times 8:30, 10:00, 11:00 and 13:00 [30].

Urine samples, collected in healthy subjects daily in the morning over one month showed an intra-individual variability, which, from the set of seven markers, was the highest for 8-isoprostane and lowest for 8-OHdG levels, denoted as the most suitable biomarker for spot urine samples, normalized for creatinine concentration [53]. Accordingly, a study by Barregard et al. found that the diurnal variability in 8-OHdG concentration for creatinine-adjusted results was a maximum of 6%, and no statistically significant impact of time was observed. However, the intraindividual variability between different days in 8-OHdG morning excretion was approximately 20% for creatinine-adjusted first void samples [54]. Therefore, we cannot completely exclude the possibility of circadian effects in this study, which may influence the results for all subjects.

Importantly, we can exclude circadian variation as the main cause of elevation of oxidative stress markers in the workers handling nanoparticles. The main argument to support this conclusion is the fact that in our studies in nano-TiO_2_ workers and nano-Fe-oxides workers, the collections of the pre-shift and/or post-shift samples were always carried out at different times of day and night over 24 h, as they worked in three 8 h shifts [32,33]. The circadian variation can, thus, be ruled out.

- Environmental contamination

Environmental air contamination may cause inflammatory or immunological responses in the airways, which may alter EBC composition [15]. In addition, markers in other bodily fluids may be affected, and the levels of MDA in the EBC and urine have been used as biomarkers of air pollution-induced oxidative stress [55]. Such nanoparticles originating from iron-containing combustion and friction were detected in the nuclei of brain cells, with reduced DNA integrity in young deceased residents of highly air-polluted cities [56]. In our previous studies, we did not confirm the effects of air pollution [13,14]. In this study, the environmental air pollution measured in the area was low, and no consistent positive correlation of these contaminants with oxidative stress markers was found.

## 5. Strengths and Limitations

To our knowledge, this is the largest study of workers handling nanomaterials, analyzing by highly sensitive methods the markers of oxidative stress in three biological fluids—EBC, plasma, and urine. This was carried out over two consecutive years. In addition, the same EBC markers were available for the workers of the same plant from 2016, as they had already been examined.

The new results of this study, for both pre-shift and post-shift measurements, showed a similar trend in all biological fluids used during the three-year study, which supports the value of the noninvasive collection of biomarkers in the EBC.

One of the limitations is the size of our groups of workers and control subjects we could collect data from. This may be the reason why the differences did not display significance in certain types of samples and time. Unfortunately, the number of employees is limited, which is a common problem in plants handling nanocomposites. Another persisting problem is the low willingness of management at plants handling engineered nanoparticles to participate in such studies, as they may potentially result in various legislative impacts and limitations [57,58].

The static measurement may not reflect the real situation in the workshop, where the workers carried out different tasks. Some workers were closer to the machines, while others were more distant. The space and the distance in machining workshop 2 were larger than in workshop 1, where the workers had to be closer to the welding machines. This disparity could be solved by personal samplers of nanoparticles, which the workers could test for the first time in 2019 and 2020.

The main limitation is the unexplained elevation of 50% of the markers of oxidative stress in the control subjects in 2018. No individual characteristics from the questionnaire positively correlated with the markers studied in a consistent way and did not cause significant differences due to their potentially weaker effect. Importantly, all post-shift results in the workers were still significantly higher than afternoon markers in the control subjects.

A possible explanation of afternoon elevation of marker levels in the control subjects may be due to the combination of several individual factors, such as physical activity in between the two collections of biological samples, like spending more energy walking home or traveling through the city center, using local transportation for lunch and then again after returning to the plant. The diet of the control subjects during lunch may have been different from the canteen in the research plant and could potentially result in some differences. A combination of these factors and their random occurrence may have played a role. An attack of a viral disease in one control subject, which was reflected by four positive correlations of the markers, may also have played a role. According to some studies, sleep and mood may also impact the concentration of oxidative stress markers.

Under an extremely controlled environment, we would expect the variability of biomarkers to decrease; however, the applicability of such research to real-world scenarios would be limited.

## 6. Conclusions

It has been experimentally proven that oxidative stress due to nanomaterials exposure leads to inflammation, cell apoptosis and genotoxicity, and a chronic low-dose exposure also causes cellular reprogramming, disease initiation and other systemic damage [12,59]. Occupational medicine is facing a shortage of studies of biomarkers of effects related to exposure to nanomaterials for subjects with the highest exposure [60,61].

This study of nanocomposites in the handling workers found that both follow-up years, the pre-shift elevations of 50.0% in EBC, 37.5% in plasma and 6.25% in the urine biomarkers agreed with the previous results. Post-shift elevation reached 62.5% in EBC, 68.8% in plasma and 18.8% in urine samples. As to the post-shift acute elevation, it was seen in the workers despite the potential interference of other non-occupational factors discussed, as a significant post-shift decrease never occurred. The working procedure in which the subjects worked was not important since a high proportion of nanoparticles was measured during all procedures in all three years.

The conclusion of the first aim of this study is that the elevation of levels of the markers of oxidative stress in nanocomposite workers stays more or less stable over three years in the EBC and does not show trends, such as cumulative effect on the one side or adaptation on the other side. These results have been supported by follow-up analysis of the plasma and urine markers. 

To obtain more significant results, a repeated collection of the post-shift samples after a longer time interval, such as 8–12 h, may be useful. In addition, individual data from the workers performing personal sampling of nanoparticles for measurement will be used in the future.

As to our second aim of the study, the answer is optimistic, as we believe that the sometimes-underestimated analysis of EBC samples proved to be a valuable method in comparison with other biological fluids studied. In addition, this approach showed identical trends for both plasma and urine, not influenced by the EBC concentration. This noninvasive method can be repeated without adverse events in short time intervals with good reproducibility [62].

The effect of inhalation of nanoparticles is not limited to the respiratory tract, as the elevation of most plasma and urine markers occurred, and a systemic effect is plausible.

Concerning our third aim of the study, at minimum, the marker that should be included in the pre-shift markers in the EBC is 8-OHdG, as this marker was significantly elevated over all three following years. For the acute exposure, MDA appeared to be the most sensitive marker, as it elevated in the post-shift EBC every year. As stated, one single test is insufficient to assess oxidative stress, and optimally, the whole set of these eight biomarkers should be used, as they reportedly reflect different types of oxidative stress [1,2,4,5].

Clearly, other not fully known factors may also play a role. The methods for the analysis of the markers are highly sensitive, with fluctuations between the years, and it is hypothesized that they respond to other stimuli, such as genetics and lifestyle, as contributing factors to the individual status of oxidative stress. Therefore, a detailed assessment of individual parameters, including personal habits, diet, physical activity, time of sampling and environmental contamination data, should always be given.

## Figures and Tables

**Table 1:** General characteristics of the groups.

	2016			2017			2018		
	Workers	Controls	p^a^	Workers	Controls	p^a^	Workers	Controls	p^a^
N (male/female)	20 (15/5)	21 (15/6)	0.796	20 (13/7)	20 (13/7)	1	21 (16/5)	18 (12/6)	1
Age (years)	42 ± 11	38.7 ± 9.1	0.334	39 ± 11	39.9 ± 7.3	0.647	40 ± 12	45 ± 13	0.253
Mean ± SD (range)	(29–63)	(20–55)		(23–64)	(27–55)		(24–65)	(21–72)	
BMI (kg/m^2^)	28.0 ± 6.2	25.0 ± 4.7	0.1	24.6 ± 4.6	26.1 ± 4.6	0.294	27.0 ± 5.3	26.5 ± 5.4	0.789
Mean ± SD (range)	(18–42)	(18–37)		(18–34)	(20–37)		(19–37)	(20–39)	
Smoking	1	4	0.169	3	3	1	3	3	0.91
Alcohol (occasionally)	18	16	0.24	16	18	0.389	18	16	0.842

N = number of subjects; SD = standard deviation; BMI = body mass index, p^a^ = exposed vs. controls within a given year.

**Table 2:** Exposure characteristics of the complete researchers’ groups for 3 years.

Exposure Time	2016	2017	2018
	Mean ± SD (Range)	Mean ± SD (Range)	Mean ± SD (Range)
Total (years)	18 ± 10 (5–40)	12.2 ± 9.3 (2–31)	13.9 ± 9.4 (1–32)
Usually per day (min)	101 ± 60 (30–240)	128 ± 87 (30–360)	123 ± 16 (60–270)
On monitoring day (min)	156 ± 62 (60–330)	204 ± 55 (150–360) **	156 ± 7.4 (120–240)
Latency since last exposure (hour)	266 ± 458 (14–1440)	81 ± 125 (15–504)	160 ± 190 (14–720)

SD = standard deviation; ** *p* < 0.01.

**Table 3:** Total mass concentration of elements subsequently analyzed by scanning electron microscope with energy-dispersive X-ray spectroscopy (SEM/EDS) during machining in the years 2016–2018.

Operation: Machining	Size Range (nm)	Sum of Elements Analyzed by SEM/EDS (µg/m^3^)
2016	25–100	0.143
2017	25–100	0.550
2018	25–100	0.801

**Table 4:** Total number concentrations of three PM fractions (nano to 10 µm) were measured by online monitoring (SMPS and APS) during the shift in individual working operations and backgrounds (medians for the respective time periods).

		Medians of Total Number Concentrations of PM Fractions per cm^3^			
	Operations and Backgrounds	<100 nm	100 nm–1 µm	1–10 µm	Total <10 µm
2016	MAG welding—workshop 1	4.88 × 10^4^	7.29 × 10^4^	3.33 × 10^1^	1.22 × 10^5^
	Smelting—workshop 1	4.60 × 10^4^	2.60 × 10^3^	3.26	4.86 × 10^4^
	Background—workshop 1	2.01 × 10^4^	6.72 × 10^2^	1.05	2.08 × 10^4^
	Machining—workshop 2	3.22 × 10^5^	2.04 × 10^5^	2.96 × 10^1^	5.26 × 10^5^
	Background—workshop 2	1.16 × 10^5^	1.68 × 10^5^	1.86 × 10^1^	2.84 × 10^5^
2017	Machining—workshop 2	8.99 × 10^4^	3.95 × 10^3^	4.75 × 10^1^	9.39 × 10^4^
	Background—workshop 2	4.68 × 10^3^	4.10 × 10^3^	6.04 × 10^−1^	8.77 × 10^3^
2018	MAG welding—workshop 1	5.52 × 10^3^	3.79 × 10^3^	1.31	9.31 × 10^3^
	Background—workshop 1	6.83 × 10^3^	2.37 × 10^3^	4.98 × 10^−1^	9.21 × 10^3^
	Machining—workshop 2	2.61 × 10^4^	3.04 × 10^3^	1.85 × 10^1^	2.91 × 10^4^
	Background—workshop 2	3.64 × 10^3^	1.68 × 10^3^	2.38 × 10^−1^	5.32 × 10^3^

SMPS = scanning mobility particle sizer; APS = aerodynamic particle sizer; MAG = metal active gas.

**Table 5:** Number proportions of particulate matter (PM) fractions measured by online monitoring (SMPS and APS) during the shift related to individual working processes.

Year	Processes	Proportion of PM Fractions (number %)		
		<100 nm	100 nm–1 µm	1 μm–10 µm
2016	MAG Welding (workshop 1)	40.13	59.85	0.02
	Smelting (workshop 1)	94.64	5.35	0.01
	Background (workshop 1)	96.76	3.23	0.00
	Machining (workshop 2)	61.23	38.76	0.01
	Background (workshop 2)	40.89	59.10	0.01
2017	Machining (workshop 2)	95.74	4.20	0.05
	Background (workshop 2)	53.30	46.69	0.01
2018	MAG Welding (workshop 1)	59.29	40.70	0.01
	Background (workshop 1)	74.24	25.76	0.00
	Machining (workshop 2)	89.50	10.43	0.07
	Background (workshop 2)	68.42	31.58	0.00

The background was measured overnight before the start of the individual working process in the workshop. PM = particulate matter, SMPS = scanning mobility particle sizer, APS = aerodynamic particle sizer, MAG = metal active gas.

**Table 6:** Mass percentage of selected elements smaller than 100 nm in the years 2016–2018.

Year		Fe	Mn	Al	Si	S	F	Na	Cl	K	Ca	Cr	Total
2016	Welding	84.70	8.46	0.00	5.35	0.61	0.23	0.44	0.20	0.00	0.00	0.00	100.00
	Smelting	80.13	7.48	0.36	4.40	1.24	0.07	2.85	1.90	1.58	0.00	0.00	100.00
	Machining	45.86	0.00	5.30	11.91	12.11	4.11	0.00	20.70	0.00	0.00	0.00	100.00
2017	Machining	0.00	0.00	21.09	40.92	15.94	0.00	16.70	5.35	0.00	0.00	0.00	100.00
2018	Machining	68.00	11.86	1.04	3.64	2.65	0.00	0.87	0.00	0.00	0.00	11.94	100.00
	Welding	40.74	3.99	0.55	2.95	1.59	28.73	4.39	0.67	8.89	7.49	0.00	100.00

Note: Mg, Ti and P were also measured; however, their proportion in the nanofraction was below their detection limit (approximately 0.1, 0.3 and 0.2 weight%, respectively) measured using a scanning electron microscope with energy-dispersive X-ray spectroscopy (SEM/EDS).

## Supplementary Materials

The following are available online at https://www.mdpi.com/2079-4991/10/12/2440/s1, Figure S1. SMPS + APS number concentration in wider size bins in 2017 related to (a) machining processes (grinding and milling) in workshop 2; (b) background to machining, Figure S2. SMPS + APS number concentration in wider size bins in 2018 related to (a) welding process (MAG) in workshop 1; (b) machining processes (grinding and milling) in workshop 2; (c) background to welding; (d) background to machining, Table S1. Mean levels of oxidative stress markers in the morning exhaled breath condensate (EBC) samples of the control group, Table S2. Mean levels of oxidative stress markers in the pre-shift exhaled breath condensate (EBC) samples of the workers, Table S3. Mean levels of oxidative stress markers in the morning plasma samples of the control group, Table S4. Mean levels of oxidative stress markers in the pre-shift plasma samples of the workers, Table S5. Mean levels of oxidative stress markers in the morning urine samples of the control group, Table S6. Mean levels of oxidative stress markers in the pre-shift urine samples of the workers.
