# Supplementary material for: Three-Year Study of Markers of Oxidative Stress in Exhaled Breath Condensate in Workers Producing Nanocomposites, Extended by Plasma and Urine Analysis in Last Two Years
Source: Nanomaterials (Basel). 2020 Dec 6;10(12):2440. doi: 10.3390/nano10122440 (PMC7762143; doi:10.3390/nano10122440)
Supplement: Supplementary file 1 [file nanomaterials-10-02440-s001.pdf]

Supplementary Materials

Three-Year Study of Markers of Oxidative Stress in Exhaled Breath Condensate in Workers Producing Nanocomposites, Extended by Plasma and Urine Analysis in Last Two Years

Daniela Pelclova [1,*], Vladimir Zdimal [2], Martin Komarc [3,4], Jaroslav Schwarz [2], Jakub Ondracek [2], Lucie Ondrackova [2], Martin Kostejn [2], Stepanka Vlckova [1], Zdenka Fenclova [1], Stepanka Dvorackova [5], Lucie Lischkova [1], Pavlina Klusackova [1], Viktoriia Kolesnikova [1], Andrea Rossnerova [6] and Tomas Navratil [7]

[1] Department of Occupational Medicine, First Faculty of Medicine, Charles University in Prague and General University Hospital in Prague, Na Bojisti, 128 00 Prague, Czech Republic; stepanka.vlckova@vfn.cz (S.V.); zdenka.fenclova@lf1.cuni.cz (Z.F.); lucie.lischkova@vfn.cz (L.L.); pavlina.klusackova@vfn.cz (P.K.); viktoriia.kolesnikova@vfn.cz (V.K.)

[2] Institute of Chemical Process Fundamentals CAS, Rozvojova 1/135, 165 02 Prague, Czech Republic; zdimal@icpf.cas.cz (V.Z.); schwarz@icpf.cas.cz (J.S.); ondracek@icpf.cas.cz (J.O.); ondrackova@icpf.cas.cz (L.O.); kostejn@icpf.cas.cz (M.K.1)

[3] Institute of Biophysics and Informatics, First Faculty of Medicine, Charles University and General University Hospital in Prague, Salmovska, 120 00 Prague, Czech Republic; martin.komarc@lf1.cuni.cz or komarc@ftvs.cuni.cz (M.K.2)

[4] Faculty of Physical Education and Sport, Charles University and General University Hospital in Prague, José Martího 31, 162 52 Prague, Czech Republic;

[5] Department of Machining and Assembly, Department of Engineering Technology, Department of Material Science, Faculty of Mechanical Engineering, Technical University in Liberec, Studentska 1402/2, 461 17 Liberec, Czech Republic; stepanka.dvorackova@tul.cz

[6] Department of Genetic Toxicology and Epigenetics, Institute of Experimental Medicine CAS, Videnska 1083, 142 20 Prague, Czech Republic; andrea.rossnerova@iem.cas.cz

[7] J. Heyrovský Institute of Physical Chemistry CAS, Dolejškova, 182 23 Prague, Czech Republic; Tomas.Navratil@jh-inst.cas.cz

* Correspondence: daniela.pelclova@lf1.cuni.cz; Tel.: +420-224-964-532

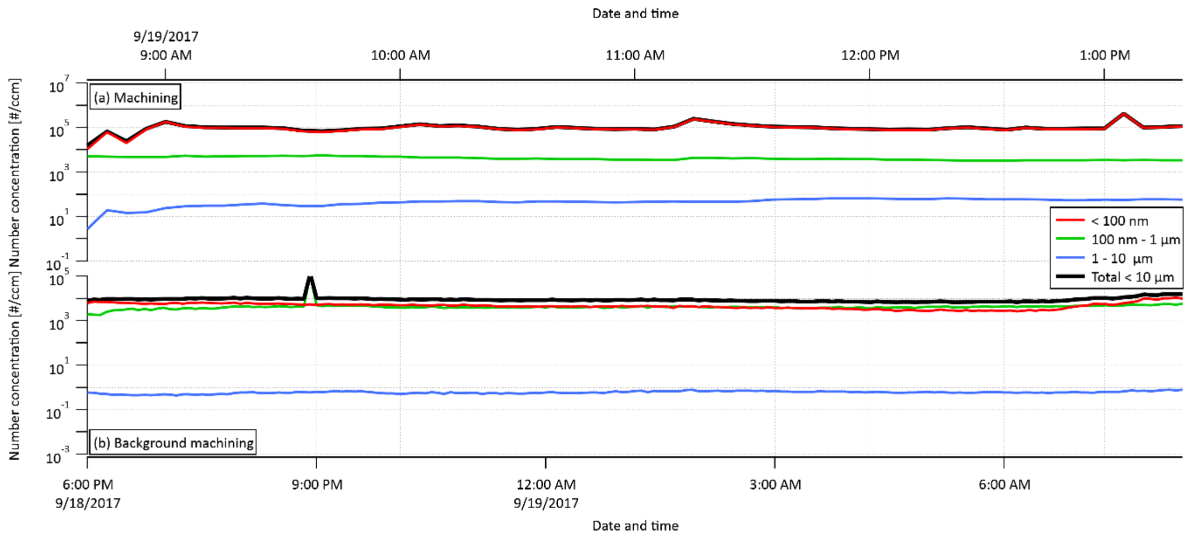

Figure S1. SMPS+APS number concentration in wider size bins in 2017 related to (**a**) Machining processes (grinding and milling) in workshop 2; (**b**) Background to Machining.

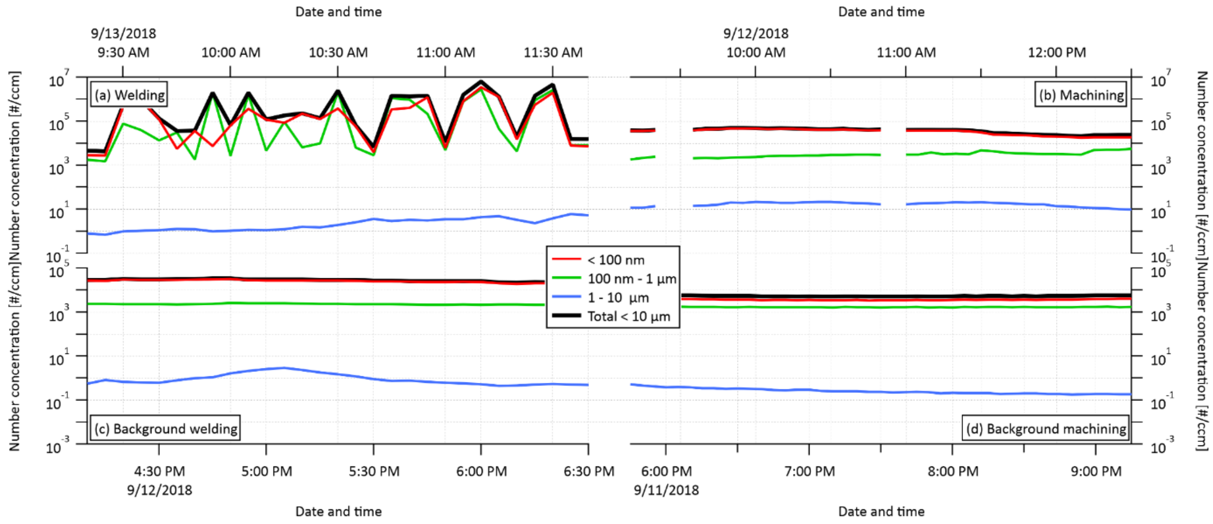

Figure S2. SMPS+APS number concentration in wider size bins in 2018 related to (**a**) Welding process (MAG) in workshop 1; (**b**) Machining processes (grinding and milling) in workshop 2; (**c**) Background to welding; (**d**) Background to Machining.

Table S1. Mean levels of oxidative stress markers in the morning exhaled breath condensate (EBC) samples of the control group.

MDA = malondialdehyde, C6–12 = aldehydes C6–C12, 8-isoprostane = 8-*iso*-prostaglandin F2*α* (8-isoprostane), 8-OHdG = 8-hydroxy-2-deoxyguanosine, 8-OHG = 8-hydroxyguanosine, 5-OHMeU = 5-hydroxymethyl uracil, o-Tyr = *o*-tyrosine, 3-NOTyr = 3-nitrotyrosine.

**Units**	**ng/mL<br>= µg/L**	**pmol/mL<br>= nmol/L**
**MDA**	18.4	255.3
**C6-12**	13.0	100.4
**Units**	**pg/mL<br>= ng/L**	**fmol/mL<br>= pmol/L**

**8-isoprostane**	20.5	58
**8-OHdG**	16.7	59
**8-OHG**	14.4	48
**5-OHMeU**	19.5	137
**o-Tyr**	21.3	118
**3-NOTyr**	27.7	122

Table S2. Mean levels of oxidative stress markers in the pre-shift exhaled breath condensate (EBC) samples of the workers.

MDA = malondialdehyde, C6–12 = aldehydes C6–C12, 8-isoprostane = 8-*iso*-prostaglandin F2α (8-isoprostane), 8-OHdG = 8-hydroxy-2-deoxyguanosine, 8-OHG = 8-hydroxyguanosine, 5-OHMeU = 5-hydroxymethyl uracil, o-Tyr = *o*-tyrosine, 3-NOTyr = 3-nitrotyrosine.

**Units**	**ng/mL = µg/L**	**pmol/mL = nmol/L**
**MDA**	18.9	262.3
**C6-12**	13.7	105.8
**Units**	**pg/mL = ng/L**	**fmol/mL = pmol/L**
**8-isoprostane**	25.9	73
**8-OHdG**	18.7	66
**8-OHG**	14.8	49
**5-OHMeU**	20.0	141
**o-Tyr**	24.0	132
**3-NOTyr**	29.4	129

Table S3. Mean levels of oxidative stress markers in the morning plasma samples of the control group.

MDA = malondialdehyde, C6–12 = aldehydes C6–C12, 8-isoprostane = 8-*iso*-prostaglandin F2α (8-isoprostane), 8-OHdG = 8-hydroxy-2-deoxyguanosine, 8-OHG = 8-hydroxyguanosine, 5-OHMeU = 5-hydroxymethyl uracil, o-Tyr = *o*-tyrosine, 3-NOTyr = 3-nitrotyrosine.

**Marker in plasma**	**ng/mL = µg/L**	**pmol/mL = nmol/L**
**MDA**	56.5	784.1

**C6-12**	74.5	589.6
	**pg/mL = ng/L**	**fmol/mL = pmol/L**
**8-isoprostane**	33.0	93
**8-OHdG**	66.3	234
**8-OHG**	69.5	232
**5-OHMeU**	59.7	420
**o-Tyr**	132.7	732
**3-NOTyr**	86.4	380

Table S4. Mean levels of oxidative stress markers in the pre-shift plasma samples of the workers.

MDA = malondialdehyde, C6–12 = aldehydes C6–C12, 8-isoprostane = 8-*iso*-prostaglandin F2α (8-isoprostane), 8-OHdG = 8-hydroxy-2-deoxyguanosine, 8-OHG = 8-hydroxyguanosine, 5-OHMeU = 5-hydroxymethyl uracil, o-Tyr = *o*-tyrosine, 3-NOTyr = 3-nitrotyrosine.

**Units**	**ng/mL = μg/L**	**pmol/mL = nmol/L**
**MDA**	59.8	829.9
**C6-12**	81.3	645.3
**Units**	**pg/mL = ng/L**	**fmol/mL = pmol/L**
**8-isoprostane**	36.8	104
**8-OHdG**	68.9	243
**8-OHG**	75.1	251
**5-OHMeU**	62.1	437
**o-Tyr**	136.0	751
**3-NOTyr**	88.5	390

Table S5. Mean levels of oxidative stress markers in the morning urine samples of the control group.

creat. = creatinine, MDA = malondialdehyde, C6–12 = aldehydes C6–C12, 8-isoprostane = 8-*iso*-prostaglandin F2α (8-isoprostane), 8-OHdG = 8-hydroxy-2-deoxyguanosine, 8-OHG = 8-hydroxyguanosine, 5-OHMeU = 5-hydroxymethyl uracil, o-Tyr = *o*-tyrosine, 3-NOTyr = 3-nitrotyrosine.

Units	µg/mmol creat.<br>= mg/mol creat.	nmol/mmol creat.<br>= µmol/mol creat.	ng/mg creat.<br>= µg/g creat.	pmol/mg creat.<br>= nmol/g creat.
**MDA**	1.8	25.0	15.9	220.8
**C6-12**	17.1	118.1	151.3	1043.7
**Units**	**ng/mmol creat.<br>= µg/mol creat.**	**pmol/mmol creat.<br>= nmol/mol creat.**	**pg/mg creat.<br>= ng/g creat.**	**fmol/mg creat.<br>= pmol/g creat.**
**8-isoprostane**	4.1	11.6	36.6	102.2
**8-OHdG**	32.9	116.2	290.8	1026.8
**8-OHG**	54.2	181.1	479.1	1601.2
**5-OHMeU**	5.6	39.4	49.5	348.4
**o-Tyr**	30.2	166.7	267.0	1473.4
**3-NOTyr**	23.3	102.6	206.0	906.6

Table S6. Mean levels of oxidative stress markers in the pre-shift urine samples of the workers.

creat. = creatinine, MDA = malondialdehyde, C6–12 = aldehydes C6–C12, 8-isoprostane = 8-*iso*-prostaglandin F2α (8-isoprostane), 8-OHdG = 8-hydroxy-2-deoxyguanosine, 8-OHG = 8-hydroxyguanosine, 5-OHMeU = 5-hydroxymethyl uracil, o-Tyr = *o*-tyrosine, 3-NOTyr = 3-nitrotyrosine.

Units	µg/mmol creat.<br>= mg/mol creat.	nmol/mmol creat.<br>= µmol/mol creat.	ng/mg creat.<br>= µg/g creat.	pmol/mg creat.<br>= nmol/g creat.
**MDA**	1.8	24.3	15.5	214.7
**C6-12**	17.3	119.3	152.8	1054.7
**Units**	**ng/mmol creat.<br>= µg/mol creat.**	**pmol/mmol creat.<br>= nmol/mol creat.**	**pg/mg creat.<br>= ng/g creat.**	**fmol/mg creat.<br>= pmol/g creat.**
**8-isoprostane**	4.0	11.3	35.3	99.5
**8-OHdG**	35.6	125.7	314.7	1111.1
**8-OHG**	56.8	189.8	502.1	1678.0
**5-OHMeU**	4.9	34.5	43.3	304.8

**o-Tyr**	31.5	173.9	278.5	1536.9
**3-NOTyr**	24.1	106.1	213.0	937.8
